# Distribution and history of extensional stresses on vWF surrogate molecules in turbulent flow

**DOI:** 10.1038/s41598-021-04034-9

**Published:** 2022-01-07

**Authors:** Oanh L. Pham, Samuel E. Feher, Quoc T. Nguyen, Dimitrios V. Papavassiliou

**Affiliations:** School of Chemical, Biological and Materials Engineering, The University of Oklahoma, 100 East Boyd, SEC T-335, Norman, OK 73019 USA

**Keywords:** Computational biophysics, Biomedical engineering, Chemical engineering, Mechanical engineering, Computational science, Cardiac device therapy

## Abstract

The configuration of proteins is critical for their biochemical behavior. Mechanical stresses that act on them can affect their behavior leading to the development of decease. The von Willebrand factor (vWF) protein circulating with the blood loses its efficacy when it undergoes non-physiological hemodynamic stresses. While often overlooked, extensional stresses can affect the structure of vWF at much lower stress levels than shear stresses. The statistical distribution of extensional stress as it applies on models of the vWF molecule within turbulent flow was examined here. The stress on the molecules of the protein was calculated with computations that utilized a Lagrangian approach for the determination of the molecule trajectories in the flow filed. The history of the stresses on the proteins was also calculated. Two different flow fields were considered as models of typical flows in cardiovascular mechanical devises, one was a Poiseuille flow and the other was a Poiseuille–Couette flow field. The data showed that the distribution of stresses is important for the design of blood flow devices because the average stress can be below the critical value for protein damage, but tails of the distribution can be outside the critical stress regime.

## Introduction

The blood multimeric protein Von Willebrand factor (vWF) plays an important role in the primary hemostatis^1^. This high molecular weight protein could unravel from globular to elongated conformation depending on changes in blood flow at the site of a vascular injury. The vWF initiates the formation of a hemostatic plug, which can adhere to the surface of a damaged blood vessel through a binding mechanism to collagen and simultaneous binding to platelets in the blood^2^. However, the functionality of vWF is either degrated or enhanced when its molecular structure is compromised because of mechanical stresses. The resulting disease is known as the acquired von Willebrand syndrome (AcWS), which can cause blood clotting irregularities and sometimes internal bleeding, especially to patients with cardiovascular implants^3,4^. Blood pumps, ventricular assist devices (VADs) and even heart valves generate blood flow conditions that are not typical for blood flow in the cardiovascular system^5–8^. The non-physiological conditions generated as blood flows through mechanical devices (including the generation of turbulence) lead to red blood cell (RBC) damage, known as hemolysis, because of shear and extensional stresses^9–14^ and to changes of the vWF protein^15–20^.

Both shear and extensional stresses can affect the configuration of a large molecule, like the vWF molecule. Strong shear stresses can even lead to cleavage of the protein. In equilibrium the vWF maintains a globular form, but under stresses, as blood flows through a wound or a cut, the A1 and A2 domains of the protein unfold to expose them to the platelets’ GPIB-α and integrin αIIbβ3 receptors^18,21^. This process initiates the clotting. Further increase of stress leads to cleavage at the A2-A3 domain by ADAMTS-13. If stresses are applied during blood flow away from a wound, then the vWF conformation changes might lead to clotting. Such abnormal behavior can be observed in turbulent flows more than laminar flows^21^. While important, the extensional stresses are often not given equal treatment in the literature for vWF. The reason might be the fact that shear stresses are more predictable than extensional stresses in a turbulent flow field, especially the shear stress at the solid boundary of a pipe or duct that can be calculated from a force balance and knowing the pressure drop in the device. However, extensional stresses can be damaging to vWF molecules at levels that may be one order of magnitude smaller than shear stresses^10^. Further, the detailed measurement of stresses in a hemodynamic velocity field is difficult, especially in-situ for turbulent flow conditions, since it involves the measurement of derivatives of the blood velocity in all three space directions, and in every location of the flow (not only the solid walls^9^). In such cases, the use of computational methods has become critical for both the probing of the hemodynamic conditions and for devise design^22^. Results of computations, however, might depend on the numerical approach. In fact, the Food and Drug Administration (FDA) has issued challenges to the computational fluid dynamics (CFD) community for the accurate prediction of blood flow in typical blood flow devices^23–25^. In these selected devises, experimental verification was made available through different laboratories across the US. High fidelity simulations, instead of turbulence modeling that is based on Reynolds Averaged Navier–Stokes (RANS) equation approaches, can provide detailed and accurate numerical predictions while avoiding empiricisms and approximations. Canonical flows that can be used as models for blood flow can be simulated with direct numerical simulations (DNS) that avoid any empiricisms, while flow in the complicated geometries of medical devices can be simulated with the less accurate RANS techniques.

The purpose of the present study is to investigate the magnitude of extensional stresses on vWF molecules using DNS in canonical flows. Using RANS for the calculation of extensional stresses locally in a turbulent flow field would not be feasible, since RANS provides the average velocity and not the fluctuating velocity field in 3D. In addition to the flow simulation, the trajectories of particles that serve as surrogates of the vWF protein were modeled, and the values of the extensional stress along the individual vWF trajectories were calculated as they traveled in the flow field. Two flows, a plane Poiseuille flow (PF) and a plane Poiseuille–Couette flow (PC) were examined. The choice of the PC flow was made in order to examine a flow regime that is relevant to VADs, where rotating parts contribute a Couette character of the flow and the pressure drop contributes a Poiseuille flow character to the flow. The structure of a Couette flow is such that larger coherent flow structures occur and it is different than the structure of a Poiseuille flow, which corresponds to flow in blood pumps with fully turbulent flow. The trajectories of 800,000 and 500,000 vWF molecules released at different locations in the PF and PC flow fields, respectively, were calculated. The history of the particles was thus computed, allowing the calculation not only of the average value of the stresses, but also of the statistical distribution of the stresses as a function of time and as a function of the location of vWF release, and of the history of the stresses as a function of time of exposure in the flow field. The contributions of this manuscript are (1) the development of a numerical methodology for obtaining the history of the stresses on vWF molecules as they move in a flow field; (2) the detailed calculation of extensional stress statistical distributions on the vWF molecules showcasing the importance of the tails of the distributions in addition to average values; and (3) the investigation of the importance of the flow field configuration and of the location of vWF injection on the distribution of stresses over time. The results are provided in dimensionless units, while typical flow parameters are provided to relate the dimensionless stresses to actual stresses in cardiovascular devises. A person who designs a devise can transform the dimensionless quantities to actual dimensional stresses.

## Background

While there have been many papers focused on the effect of shear stress on vWF damage and blood cell trauma, the extensional components of the viscous stress also play a significant role in inducing cell damage^11,26^. The critical value of shear stress for vWF deformation is around 5 Pa^16^. It has been found that the vWF is hemostatically active only under high shear rate, *γ*, and the value of the critical activation threshold is $\gamma_{crit} \approx 5000\,{\text{s}}^{ - 1}$^27,28^. In addition, there is a sharp, well-defined, large scale extension of vWF with end-to-end lengths up to 10 μm at shear rates > 5000 s^−1^ (corresponding to shear stress ∼4–5 Pa)^29^. Investigation of vWF in solution at shear stress of 6 Pa has showed that high shear stresses and long exposure times caused loss of high molecular weight multimers of vWF, resulting in poor binding to platelets and subendothelial collagen^29^.

For blood cells, both the shear and the extensional stresses combine to act on the cell^11,14,26,30,31^. Interestingly, RBCs deform at about 20 Pa in a simple shear flow, while in an extensional flow, the deformation is slightly higher (as measured by the deformation index) and occurs at much lower stress levels (about 6 Pa). Hence, the deformation of RBCs is more efficient in an extensional flow than in a simple shear flow^10^, and one could examine the hypothesis that this could be the case for proteins^3,16^. Further, the time of exposure of a cell in a stress field affects the level of cell trauma^25,31–36^. For RBC hemolysis, power law models are often used to quantify the expected hemolysis by considering both the stress level and the time of exposure,

1 $$HI = C\tau^{\alpha } t^{\beta }$$

where *HI* is the hemolysis index (a measure of hemolysis), *τ* is shear stress, *t* is length of exposure time to stress *τ*, and the constants *α*, *β*, and *C* are obtained through experiments.

Apart from RBCs, there have been other microparticles that are affected by extensional stress in a flow field, such as synthetic, liquid filled capsules that are suspended in hyperbolic extensional flow. Experimental results have demonstrated that capsule deformation is a function of the strain rate for an extensional flow generated in a four-roll mill^37^. Other results have provided information to the extent that entomopathogenic nematodes were damaged during flow through an abrupt contraction, a predominately extensional flow regime^38^. In a combination of transient, extensional, and shear flow experimental studies, cultures of Chinese hamster ovary (CHO) showed cell damage above a critical value of turbulent kinetic energy dissipation rate^39^. Microfluidic sensing of the mechanical cell damage under an extensional stress field showed that the CHO cells were mechanically damaged when the extensional stress was greater than ∼250 Pa^40^. The use of a power law model to describe the vWF deformation appears to be justified because in these cases, where stresses affect the properties of a material, the effect depends on the duration of the stress application and the level of stress. In addition, prior research with vWF degradation with time, for example Zhussupbekov et al.^41^ and Kania et al.^2^, have indicated that a power law model applies for the unfolding of vWF under stress. Indirect evidence that a power law applies for the change in protein conformation vs time and shear rate is also offered in Heidari et al.^42^, where a normalized radius of gyration of the vWF was plotted as a function of the product of shear rate and a characteristic time. Even though the mechanisms of protein elongation and the mechanism of ADAMTS-13 cleavage are complex, applying a power law as a first order approximation to model the effects of exposure time on vWF deformation appears to make sense. The values of the exponential constants, however, are not known.

Turning the focus back on vWF, when arterioles are cut, both the shear and elongational flow components increase with the elongational flow component selectively increasing at the site of rupture. After that, the flow would decrease with subsequent vasoconstriction that leads to a decrease of shear rate. Meanwhile, the elongational flow would increase precisely at the site of vasoconstriction hence it plays an utmost important role in hemostasis^16^. Therefore, studying the behavior of vWF under extensional flow is quite necessary since there are strong indications that unraveling the multimer vWF is more impactful in extensional flow than in pure shear flow. Table 1 is a collection of data from the literature regarding the critical elongational stress, or the critical elongational rate of strain for changes in the configuration of vWF and for selected types of cells.

**Table 1:** Literature review of critical shear and extensional stresses for the deformation of vWF, cells, and capsules.

Type of deformable object	Critical shear stress or rate of strain	Critical extensional stress or rate of strain	Literature source
von Willebrand Factor (vWF)	Shear stress > 20 dyn/cm^2^		Lancellotti et al.^1^
vWF		Elongational strain rate < 300 s^−1^ no unraveling Elongational strain rate 300 to 400 s^−1^, metastable unraveling Elongational strain rate > 400 s^−1^ unraveling	Kania et al.^2^
vWF fibers	Critical shear rate = 5000 s^−1^		Schneider et al.^28^
vWF	Critical shear rate = 5522 s^−1^		Zhussupbekov et al.^41^
High molecular weight multimers of vWF	Shear rate ≥ 5000 s^–1^ for conformational changes Shear stress < 1012 dyne/cm^2^, no molecular degradation Shear stress > 3070 dyne/cm^2^ exposure time of 60 s, 90.7% molecular degradation, while exposure time of 600 s led to 96.1% molecular degradation		Jhun et al.^79^
vWF (~ 50 nm typical vWF dimer)		Critical elongation rate 300 to 600 ~ s^−1^	Sing et al.^3^
Tethered von Willebrand Factor	Critical shear rate = 5000 s^−1^ Critical shear stress = 2.0–2.5 Pa		Wang et al.^83^
Red blood cell (RBC)	Shear stress > 20 Pa	Extensional stress > 6 Pa	Lee et al.^10^
Chinese hamster ovary (CHO) cells		Extensional stress > 250 Pa	Bae et al.^40^

## Methods

Blood flow in medical devices can be Poiseuille flow (PF) or Poiseuille–Couette flow (in certain parts of a VAD or a blood pump). Therefore, this study focuses on both kinds of flow in the canonical setting of a channel. We chose the dimensions for the computational box of PC flow and the fully turbulent PF as 30*πh* × 2*h* × 4*πh* and 16*πh* × 2*h* × 2π*h*, respectively, in the *x* (streamwise), *y* (wall normal) and *z* (spanwise) directions. In the streamwise *x* and the spanwise *z* directions, the flow was assumed to be periodic and the no-slip boundary conditions were used at the channel walls. The algorithm used herein was developed by Lyons et al.^43^ to simulate flow in a channel by solving numerically the Navier–Stokes (N–S) equation. The code was for Newtonian fluids that have constant physical properties, and it has been modified by Papavassiliou^44^ to simulate Couette flow and has been extended by Kontomaris^46^ to simulate the transport of particles in the Lagrangian sense. The algorithm has been described in detail in^45^ and in the Dissertation by Lyons^46^. The results have been validated with experiments and with other DNS and experimental results for channel flow and plane Couette flow^47–49^, as well as for particle tracking in the DNS^49–51^. In this algorithm, the rotational form of the dimensionless N–S equations was solved. The equations were made dimensionless using the viscous wall parameters, i.e., the friction velocity u* (defined as $u^{*} = \left( {\tau_{w} /\rho } \right)^{1/2}$, where $\tau_{w}$ is the shear stress at the wall and *ρ* is the fluid density), the viscous length scale l^*^ = (*ν*/u^*^) and the viscous time scale t^*^ = l^*^/u^*^, where *ν* is the kinematic viscosity of the fluid. In the rotational form of the Navier–Stokes, the inertia term is replaced with the vector identity

2 $$u \cdot \nabla u = - u \times \omega + \frac{1}{2}\nabla \left( {u \cdot u} \right)$$

where *u* is the velocity vector, and the vorticity vector is $\omega = \nabla \times u$.

The time integration was done using the pseudo-spectral fractional step method developed by Orszag and Kells^52^ with a pressure step field correction suggested by Marcus^53^. The velocity field advanced from time step N to step N + 1 by three fractional steps: in the first, the non-linear convective term and the mean pressure gradient were calculated. The second fractional step calculated the dynamic pressure head and the third fractional step solved for the viscous terms of the N–S. The convective term and the mean pressure terms were advanced in time by a second order accurate semi-implicit Adams–Bashforth–Crank–Nicholson scheme. The dynamic pressure terms and the viscous term were advanced in time with a first order accurate Euler scheme. The convective (nonlinear) term was evaluated in physical space and, thus, aliasing errors occured, which were reduced by using the two-thirds truncation rule. The velocity field was expanded in terms of truncated Fourier series in the *x* and *z* directions and Chebyshev polynomial series in the wall normal direction. The periodic boundary conditions in *x* and *z* were thus satisfied with the use of Fourier series expansions. The number of Fourier modes depended on the simulation and it was equal to the number of mesh points in the *x* direction and the *z* direction. The number of Chebyshev nodes was equal to the number of mesh points in the wall-normal direction (direction *y*).

In the PC channel, the bottom wall moved in the negative *x* direction and the top wall moved in the positive *x* direction. Both walls had the same velocity, equal in magnitude to 7.78 viscous wall units. The computational boxes of the PC flow and PF were divided into 512 × 128 × 128 and 1024 × 256 × 128 mesh points, respectively. The time step for simulations was Δ*t* = 0.075 for the PC flow and Δ*t* = 0.1 for PF. The blood was assumed to be a Newtonian fluid, given the high shear rate of the flow simulations^30,54,55^. In fact, calculations for non-Newtonian fluids for the FDA blood pump did not show significant differences in stress levels for that flow field^25^. Based on the data presented in Table 1 (refs^2,3^ that indicate critical extensional shear rate between 300 s^−1^ and 400 s^−1^) the value of 350 s^−1^ for the shear rate was selected, which was combined with the blood viscosity value used here to provide the critical extensional stress value for vWF configurational changes to be 1.2 Pa. Therefore, the characteristic stress in this study is for the unraveling of the protein, and not for the ADAMTS-13 cleavage.

The friction Reynolds number *Re*_*τ*,_ for the PC flow and the fully turbulent PF was 80 and 300, respectively. In order to provide a sense of the dimensions of the simulation setups, the PC channel width was chosen to be 1.5 mm for *Re*_*τ*_ = 80, the blood viscosity $\mu = 0.0035$ Pa s, so that the value of the wall shear stress $\tau_{w}$ was 35.16 Pa corresponding to friction velocity u^*^ = 0.183 m/s,. This is a case of flow within the range of operation of VADs^56–59^. A typical flow field based on the FDA critical path initiative conditions for blood flow in the suction side of a centrifugal pump was chosen for the PF simulations^58,60–63^. For the case of *Re* = 3661 in a 1.5 mm diameter pipe and a flowrate of 7 lt/min, the corresponding mean velocity is 2.015 m/s. Comparing this velocity to the calculated dimensionless mean velocity of 16.68 in viscous wall units for the channel flow, the friction scales were calculated as u^*^ = 0.1208 m/s and $\tau_{w}$ = 14.95 Pa for *Re*_*τ*_ = 300. The DNS algorithm used was an in-house developed DNS that was based on the pseudo-spectral method of Lyons et al.^43^ which has been verified and used in our laboratory for PF, PC flow and plane Couette flow^64–66^.

The trajectories of each vWF molecule were modeled using a Lagrangian approach, i.e., Lagrangian scalar tracking (LST)^48,65,67,68^. The markers were passive and were assumed to be massless (that means that drag effects were not taken into account, and that the presence of the particles did not affect the fluid flow), so one-way coupling between the flow and the motion of the particles was employed. For particles that are assumed to be surrogates of molecules, the main driving force for marker movement is the convection effect (i.e., the velocity of the fluid) and the Brownian motion that depends on the hydraulic diameter of these particles.

These markers were released into the simulated flow field from specified locations at one instant of the simulation (instantaneous source). The convective part of the marker motion was calculated assuming that using the fluid velocity is the same as the Lagrangian velocity *V* at the particle position and integrating in time. The Brownian motion was simulated by imposing a random jump at the end of each convection step^45,69,70^. This random jump took values from a normal probability density function that had a zero mean and a standard deviation that depends on the fluid properties and the molecular diffusivity of the particles. The equation of motion for each marker in each space direction *x* is given by^71^

3 $$x_{t + 1}^{p} = x_{t}^{p} + \mathop \smallint \limits_{t}^{t + 1} V_{t}^{p} dt + Z\sigma$$

where $x_{t + 1}^{p}$ is the displacement of the marker relative to its source at time *t* + 1, $V_{t}^{p}$ is the velocity of the fluid in the *x* direction at position $x_{t}^{p}$, $\Delta t$ is the time step, and *Z* is a random number following a standard normal distribution.

A large number of mass markers were released in the flow field that was generated computationally by the DNS. The vWF markers were injected instantaneously at selected distances, Y_0_, from the bottom wall of the computational channel. The values of Y_0_ were 0, 3, 5, 15 and 80 for the PC flow, and, Y_0_ = 0, 1.5, 3, 5, 10, 15, 75, 300 for the PF (Y_0_ was measured in wall units away from the bottom channel wall). These values were selected in order to observe the changes in the distribution of the extensional stresses for markers released at the center of the channel, at the channel wall, within the viscous wall sublayer and at the buffer region. At each Y_0_, markers were released from 20 lines spanning the *x–z* plane so that possible bias caused by the initial velocity field would be avoided. Starting at x = 0, these lines were uniformly spaced in x and were spanning the width of the channel in the z direction. We injected 100,000 markers uniformly in the z direction for each value of Y_0_ (i.e., 500,000 total particles for the PC flow and 800,000 total markers for the PF). Since the markers were passive, the presence of one did not affect the trajectory of any other marker. The *x* location for each marker was calculated by subtracting the initial position *x*_0_ of the marker, therefore (x- *x*_0_) was considered as the marker location in the streamwise direction. In vector form, the location of each marker in the Lagrangian sense, $\vec{X}\left( {\overrightarrow {{x_{0} }} ,t} \right)$, represented the location of the marker that was released at position $\overrightarrow {{x_{0} }}$ at time t = t_0_ = 0, and the corresponding Lagrangian velocity of the marker at that location was $\vec{V}\left( {\overrightarrow {{x_{0} }} ,t} \right)$. Assuming that the velocity of each marker is the Eulerian velocity, $\vec{U}$, of the fluid at its location, the Lagrangian velocity was given as $\vec{V}\left( {\overrightarrow {{x_{0} }} ,t} \right) = \vec{U}\left[ {\vec{X}\left( {\overrightarrow {{x_{0} }} ,t} \right),t} \right]$. The equation of particle motion (Eq. 3) was integrated using an Adams–Bashforth scheme (explicit, second-order accurate) in each space direction. By using a mixed sixth-order Lagrangian-Chebyshev interpolation scheme to calculate the velocity vector between grid points, it was possible to track mass markers^67,69,72^. The random molecular diffusion movement of each marker was calculated at the end of the convective motion, as seen in Eq. 3. The particle displacement was selected randomly from a normal statistical distribution and following Einstein’s theory for Brownian motion. The random displacement had a mean value of zero. The standard deviation, $\sigma$, depended on the diffusivity of the vWF particle and it was calculated as $\sigma = \sqrt {2\Delta t/Sc}$, in each space direction, where Δ*t* is the time step of the simulation and *Sc* is the Schmidt number, *Sc* = *ν*/*D*. Finally, *D* is the molecular diffusivity of the vWF in blood. Assuming 1.8 × 10^–8^ m for the radius of a vWF molecule with molecular weight of 20,000KDa^73^ in a fluid with the dynamic viscosity of blood (0.0035 Pa s), the Stokes–Einstein relationship produced a diffusivity of 3.6119 × 10^–12^ m^2^/s for the vWF, and a Schmidt number of 914,000 (the blood kinematic viscosity was taken as *ν* = 3.30189 × 10^–6^ m^2^/s). This is a simplification of the vWF molecular behavior, since our algorithm does not account for different molecules that can interact and adhere to one another. However, a change in the mass marker due to molecular adhesion would only result in a change in its size, within the framework of the present calculations, leading to a change in the *Sc* that would change the trajectory of the new molecular cluster slightly, but not the stresses felt by it.

In the above, the proteins were assumed to be in the globular shape. While the shape of vWF is dynamic and dependent on the type of flow, in general the vWF multimers have overall compact, bird’s nest shape. The behavior of the vWF as a sphere has been adopted by others, e.g., Gogia et al.^29^ and Sharifi and Bark^74^. It was treated implicitly as a sphere in Pushin et al.^75^, since the forces acting on the vWF were assumed there-in to be found as stress multiplied by the area of a circle. When the hydrodynamic and mechanical stresses are low, the multimers attain a compact (collapsed) conformation because of effective interdomain attractions^76^. Above a critical shear stress, the vWF free in flow would elongate and tumble, and it would extend in elongational flow^41,74^. Since such changes were not modeled in this study, the hydrodynamic radius was used for computations.

The extensional stresses were calculated in dimensionless form as follows:

4 $$\sigma_{xx} = 2\frac{\partial u}{{\partial x}},\sigma_{yy} = 2\frac{\partial v}{{\partial y}},\sigma_{zz} = 2\frac{\partial w}{{\partial z}}$$

where *u*, *v*, and *w* are the fluid velocity components in the *x*, *y* and *z* directions respectively. This stress was calculated at the position of each vWF marker at each time step, based on interpolation of the Eulerian velocity field at the marker location. A sixth order Legendre interpolation scheme in the *x* and *z* directions and a Chebyshev polynomial interpolation in the wall-normal direction were implemented to obtain the velocity at the markers’ location^67^.

The time integration of the stresses along the marker locations to obtain the Lagrangian history of the stresses was conducted with a simple trapezoid rule since the time step of the simulations was constant, and assuming that the exponents *α* and *β* in a power law model for vWF degradation (similar to that in Eq. 1 for RBCs) would be on the order of one. The cumulative effect of the history of stresses acting on the particles was calculated by carrying out an integration of the elongational stress components with time along the trajectory of each particle and for the duration of the simulation. In the Lagrangian sense, the integral of the stress along each particle trajectory was calculated with time and the distribution of the stresses was then calculated as a function of time. The elongational stress acting on each particle was assumed to be the hydrodynamic elongational stress at the particle’s location. The sample used for the calculation of each probability distribution function appearing in the Results and Discussion section was the integral of the stress along the trajectories of all particles simulated until a specified time of exposure. The sample size, therefore, was 100,000. This Lagrangian integration of stresses over time has been used elsewhere in the literature for the calculation of the history of stresses acting on cells for hemolysis^25,33,77^.

## Results and discussion

### Poiseuille–Couette flow

**Figure 1:**
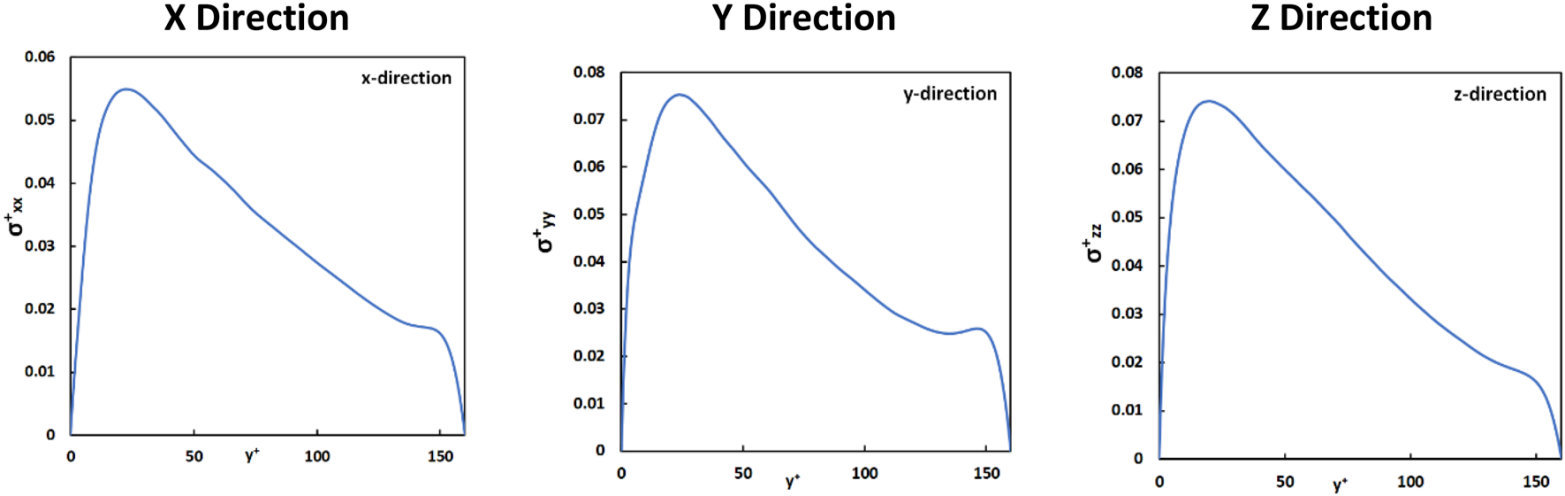
The magnitude of the extensional stress as a function of the channel height in the *x*, *y*, *z* directions in Poiseuille–Couette flow. These are averaged values of Eulerian velocity fields.

In Fig. 1 the Eulerian results for the average extensional stress profile are presented as a function of the distance from the channel wall. The stress values increased dramatically from the wall to get to a maximum value at y^+^ = 25 and then decreased gradually, before sharply falling to zero at the moving wall. One would expect that the extensional stress on vWF markers that are released in this flow field at Y_0_ < 25 would start increasing as the markers move to the regions of high extensional stress, and then decrease, as the markers move farther away into the channel. For release at the center of the PC channel, one would expect a distribution with constant mean value (as markers would move closer to the top wall as well as closer to the bottom wall), but the variance of the statistical distribution of the stresses would be expected to grow with time.

**Figure 2:**
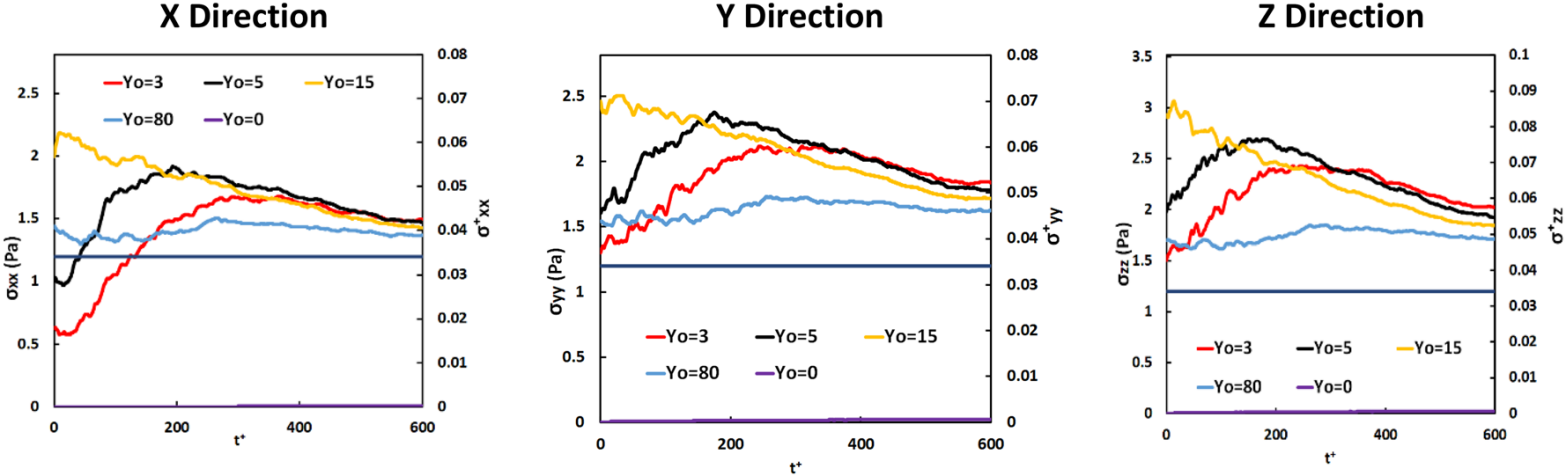
Mean of the absolute value of extensional stress for particles released at different positions in the Poiseuille–Couette flow field over time. The dark blue line represents the critical stress of 1.2 Pa for the case modeled herein.

The Lagrangian average of the extensional stresses over the vWF particles as a function of time is seen in Fig. 2. The absolute value of the extensional stress was chosen to be plotted, since the magnitude of the stress and not its direction (compressive or extensional) is the important parameter that affects the vWF molecules. The absolute values in *y* and *z* directions are larger than the critical extensional stress value (1.2 Pa). In general, the change of stress value followed the same trend in all directions. As expected, the vWF markers released close to the location of the Eulerian maximum (at Y_0_ = 15) started from a higher average value at t^+^  = 0, while those released closer to the wall were initially at smaller values. As time passed, the markers diffused away from their original location of release and eventually the average stress appeared to converge. For vWF released within the viscous sublayer (Y_0_ = 3 and 5) the stress value increased until t^+^ = 250 and then decreased gradually. The vWF markers released at the wall of the channel stayed in the wall region, since the molecular diffusion part of their motion was not enough to force them to leave the region very close to the wall. Considering the very high *Sc* for the vWF, the molecular diffusion part of the particle motion is dominated by convective effects, but close to the channel wall turbulence convection is not strong (the turbulent velocity fluctuations are rather small). The particles released at the channel center (at Y_0_ = 80) do not show significant change in the average stress over time. However, it is apparent from these figures, and specifically from the streamwise stress profile, that it is possible for vWF markers to experience lower than critical stresses as they travel in a region of the channel, and then at later times to be in regions where the stress is higher than the critical value. In that case, for small time, the proteins are functional, but at later times they might start to lose their functionality.

**Figure 3:**
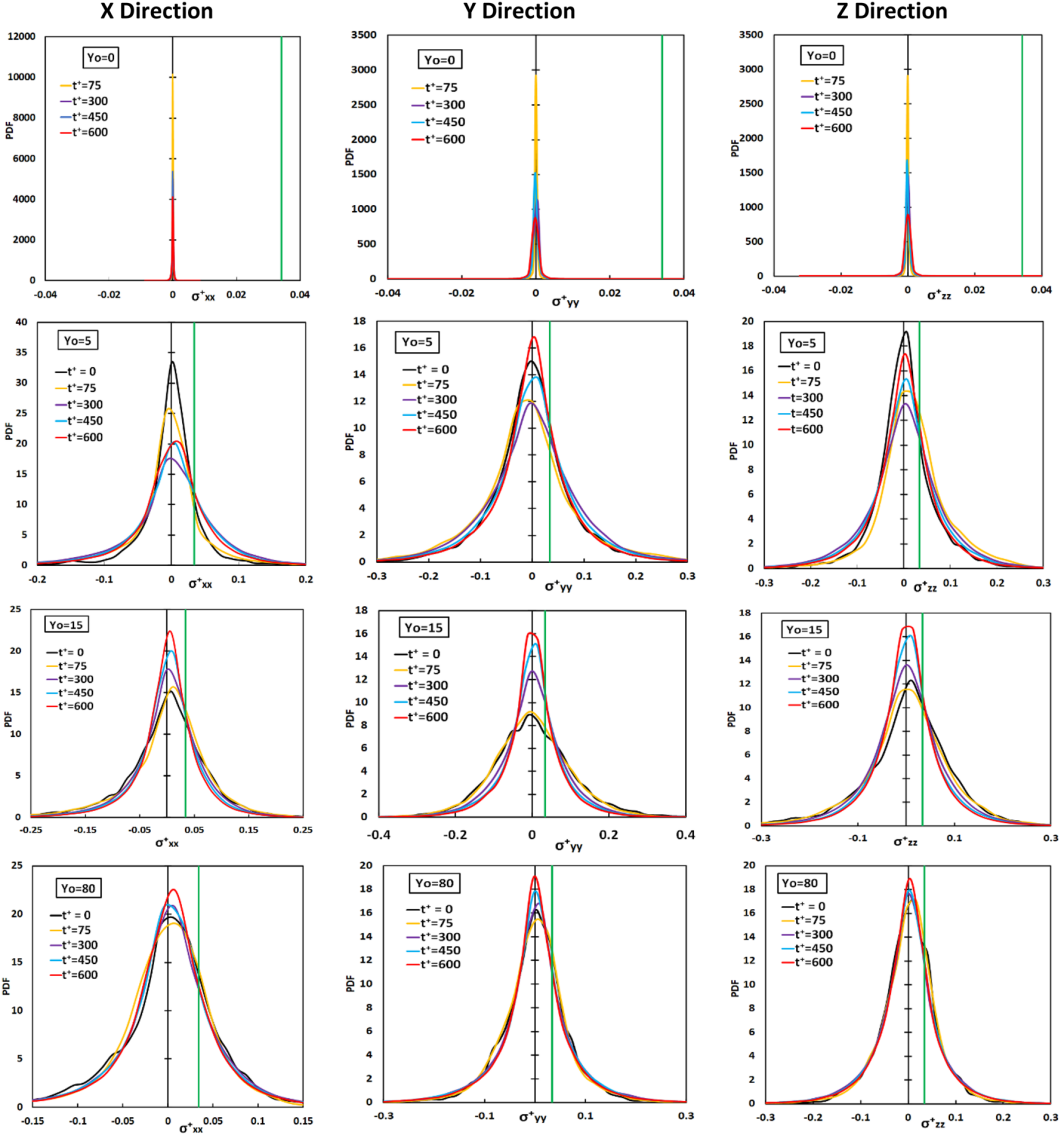
Probability density function for the extensional stress components in x, y, z directions of particles released at Y_0_ = 0, 5, 15, 80 at different time instances Poiseuille–Couette flow field. The green line represents the critical stress of 1.2 Pa for the case modeled herein.

In Fig. 3 we present the probability density function (PDF) of the statistical stress distribution in all directions as a function of time and as a function of the point of release. Releases from the wall of the channel, the edge of the viscous wall subregion (at Y_0_ = 5), the buffer region and the center of the channel were selected for presentation. For release at Y_0_ = 5, the PDF of the stresses became wider as time passed. When the release position is farther from the wall, at Y_0_ = 15, the PDF becomes narrower with time, showing that the markers were experiencing a smaller variation of stresses with time. The stress distribution remained roughly the same as a function of time for release at the center of the channel. Moreover, the PDF was symmetric for that case. The critical extensional stress is also shown on the figure for the positive stress, and it is seen that those vWF molecules that experience the stress at the right and the left tail of the distribution would be damaged by extensional stress.

**Figure 4:**
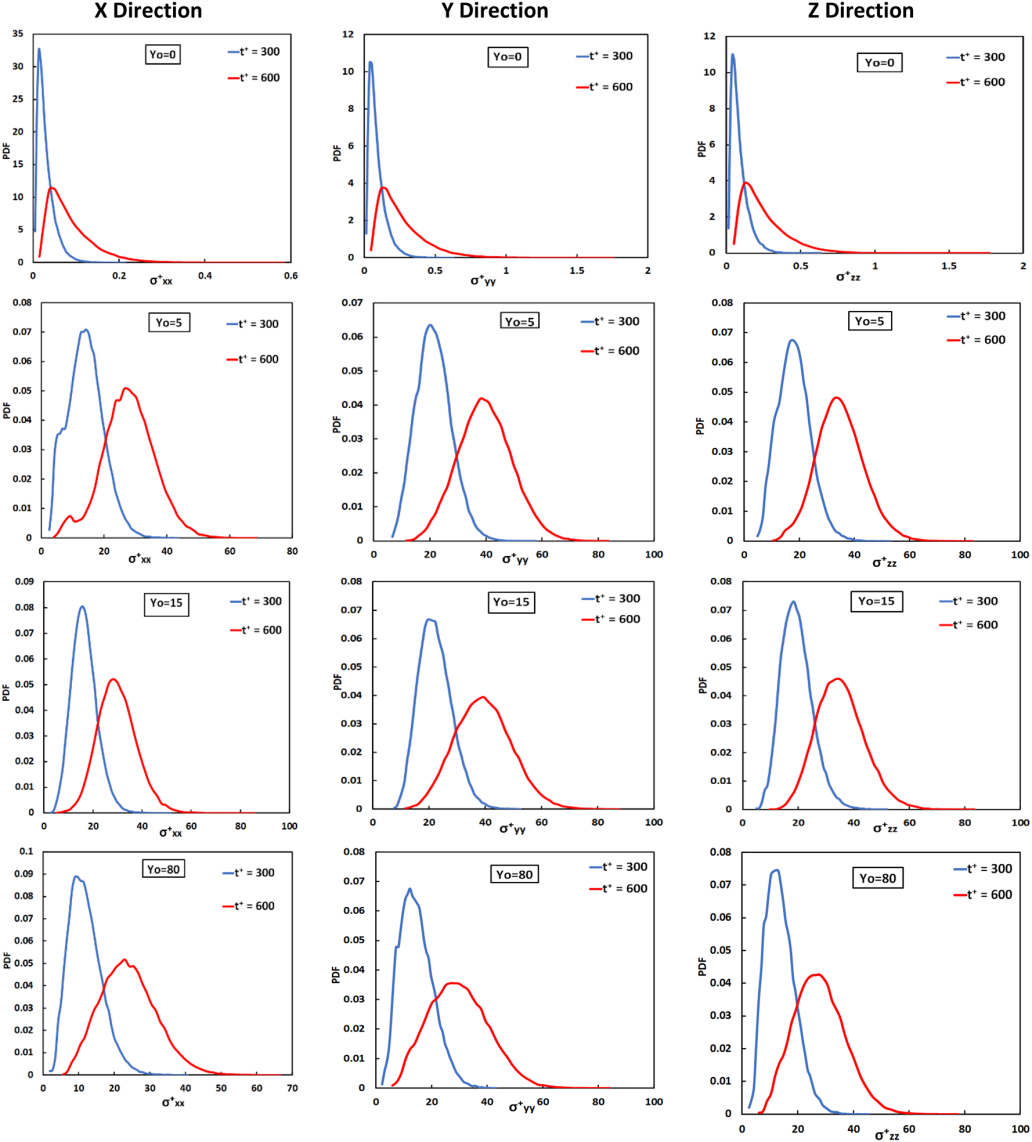
Probability density function of the extensional stress histories in x, y, z, directions for particles released at different positions at t^+^ = 300 and 600 in the Poiseuille–Couette flow field.

Figure 4 is a presentation of the PDF of the distribution of the history of the stress on particles at two time instances after being released in the flow field. According to Guria et al.^75,78^, the vWF molecule can undergo irreversible stretching or unfolding when it undergoes stresses even for small times. Therefore, the history of stresses on a molecule is important and can lead to an elongated or cleaved vWF as a result of a high stress experienced at earlier times, or as a result of low stress experienced over long times, even when the molecule at one particular snapshot in time is not stressed^75,78^. As time advanced, the distribution was displaced to larger values, since the particles experienced a stress for longer exposure times, but the variance of the distributions increased as well. The width of the distribution is an indication of the significant differences in the stress that particles underwent, and these differences seem to grow as time passed. At the release position Y_0_ = 0, the integrated stress values were much smaller when compared to the other release positions. At larger times, it appears that the stress distributions tended to be more symmetric and looked more similar to the PDF for particles released at the channel center. This is expected, since turbulent convection would mix and distribute the particles across the channel width as time passed.

### Poiseuille flow

**Figure 5:**
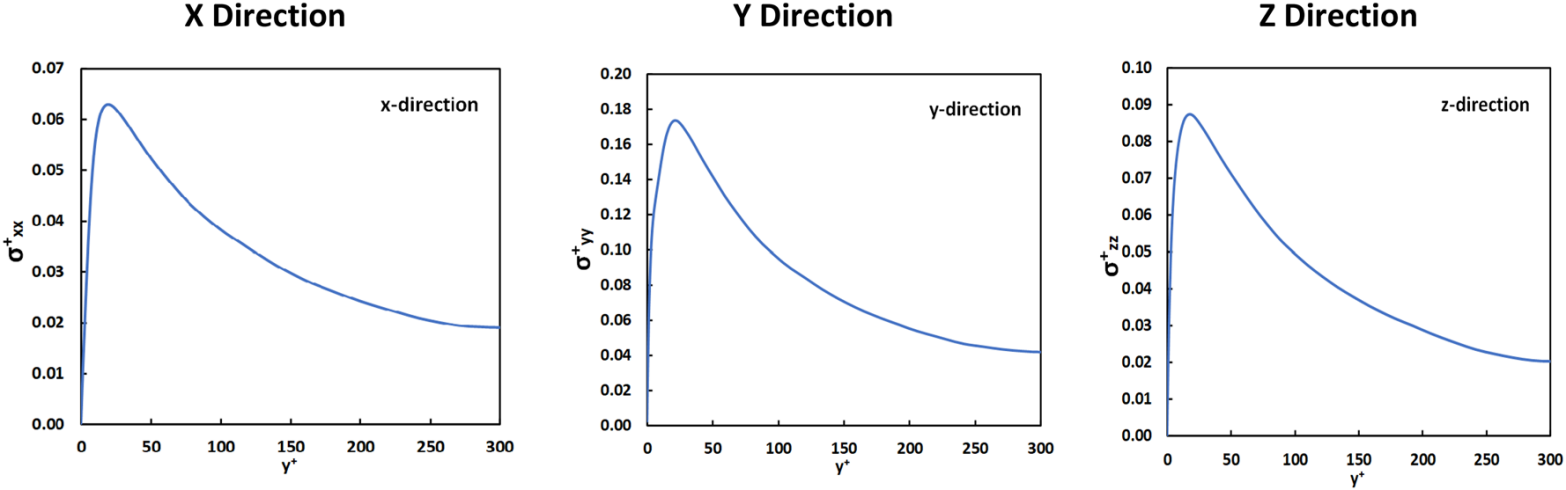
Eulerian average of the absolute value of the extensional stress as a function of the distance from the wall in plane Poiseuille flow. Since the Poiseuille flow channel is symmetric across the center place at y^+^ = 300, only the bottom half of the stress profile is presented.

The average of the absolute value of the extensional stresses in the three space directions as a function of the distance from the channel wall is presented in Fig. 5. In this case, the center *yz* plane in the channel was a plane of symmetry, so only the profile at the bottom half of the channel is shown. The stress profiles seen in Fig. 5 are Eulerian averages and they show that there was a maximum stress at y^+^ = 25 in the buffer layer. Farther out from the channel wall the stresses have lower values and reached a minimum at the center of the channel. The normal stress in the *y* direction, σ_yy_, was larger than the extensional stress in the other two directions.

**Figure 6:**
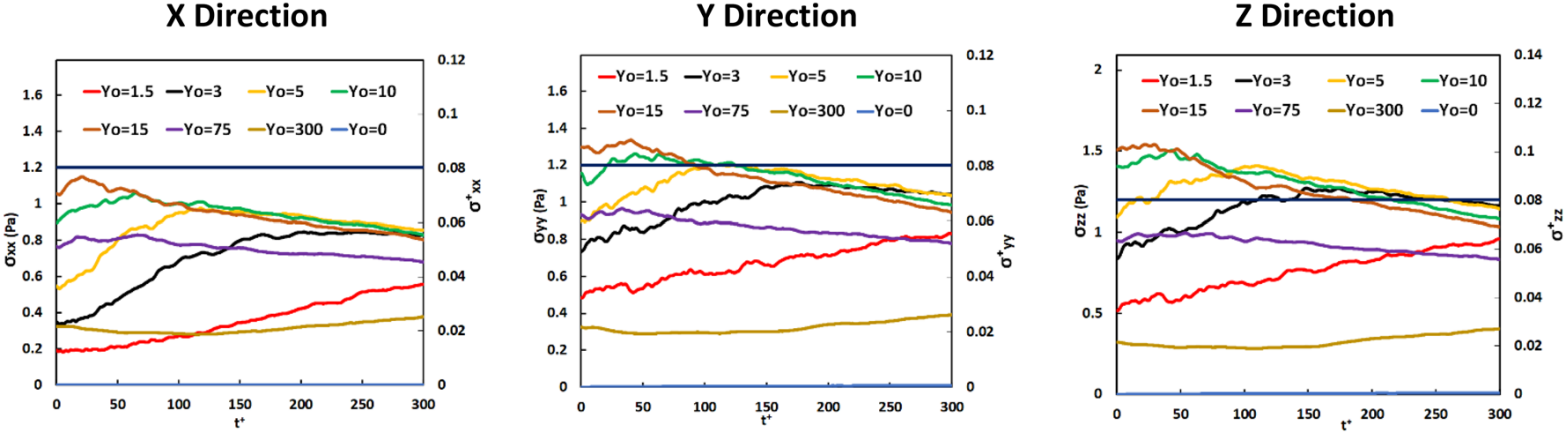
Average of the absolute value of the extensional stress on vWF model particles released at different positions in the Poiseuille flow field. The solid straight line at 1.2 Pa indicates the critical extensional stress.

Figure 6 is a presentation of the Lagrangian average of the absolute value of the extensional stresses acting on the vWF model particles as a function of time. The mean value of the extensional stress in the *x* direction, σ_xx_, was smaller than the critical value for vWF deformation. In the *y* and *z* directions, however, particles released at the edge of the viscous sublayer and within the buffer region (at Y_0_ = 3, 5 and 15) appear to have experienced high stresses on average—higher than the critical value of 1.2 Pa. In general, the change of stress value followed the same trend in all directions. The average stress for vWF particles released at the center of the channel remained roughly constant, as expected based on the Eulerian stress profile and as discussed for the PC flow case. Particles released at the wall or very close to it (at Y_0_ = 0 and 1.5) did not have a chance to disperse away from the wall and experience high stresses. As the time after particle release increased, the particles dispersed more uniformly across the channel and the average stress was expected to reach the bulk average Eulerian value across the channel.

**Figure 7:**
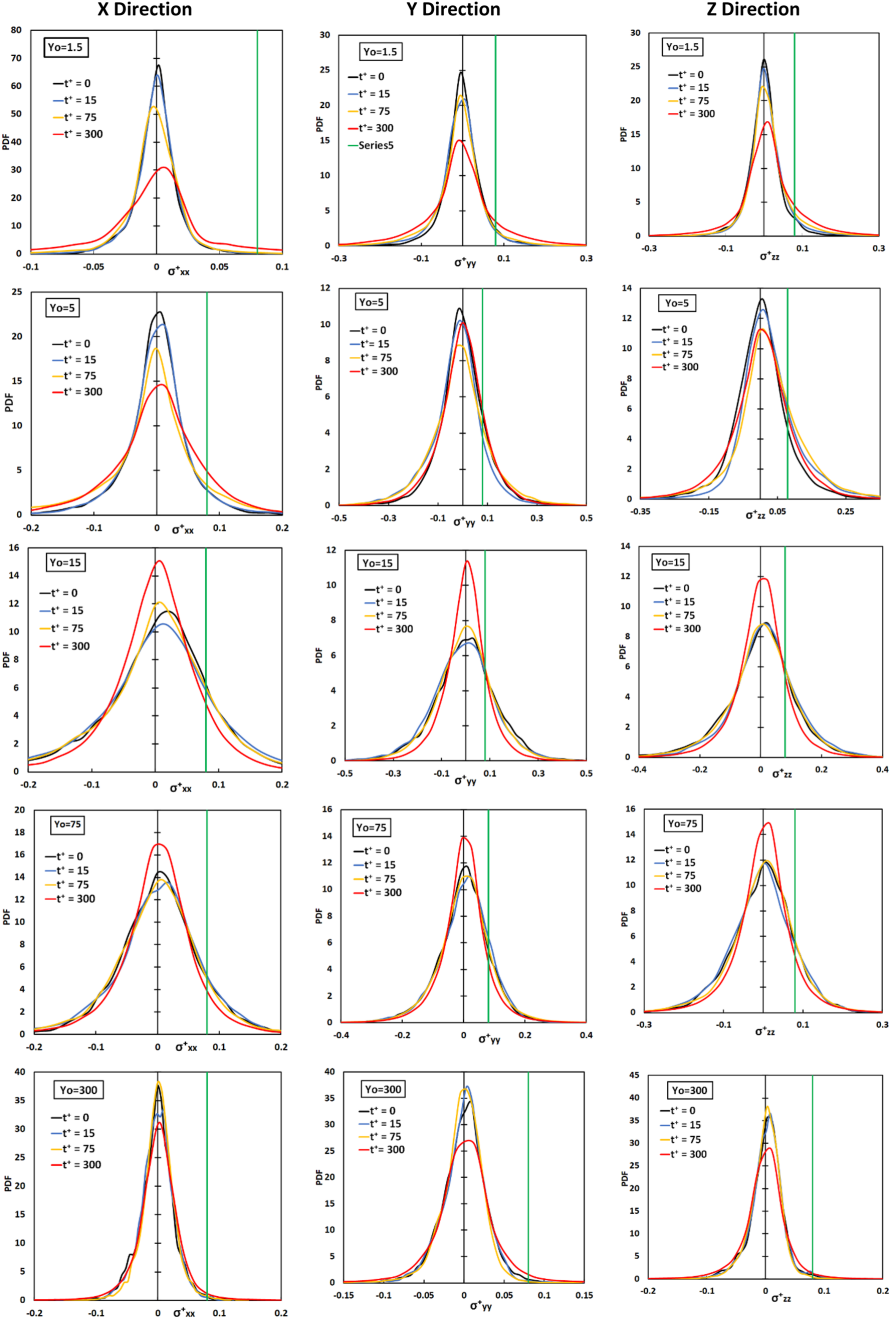
Probability density function for the extensional stress components in x, y, z directions of particles released at Yo = 1.5, 5, 15, 75, 300 at different time instances in the Poiseuille flow field. The solid green line depicts the critical stress value for vWF deformation.

After injecting particles, the full distribution of extensional stress was calculated at specific time instances and is presented in Fig. 7. It appears that for all three directions *x*, *y*, *z* the extensional stress distribution for particles released within the viscous sublayer and the buffer region changed the most with time. The most dramatic change was from t^+^ = 15 to t^+^ = 75. The variance of the PDF for the stress distribution increased as time elapsed, as indicated by the widening of the PDFs. When particles were released in the log-layer (Y_0_ = 75) and at the center of the channel the changes were moderate.

**Figure 8:**
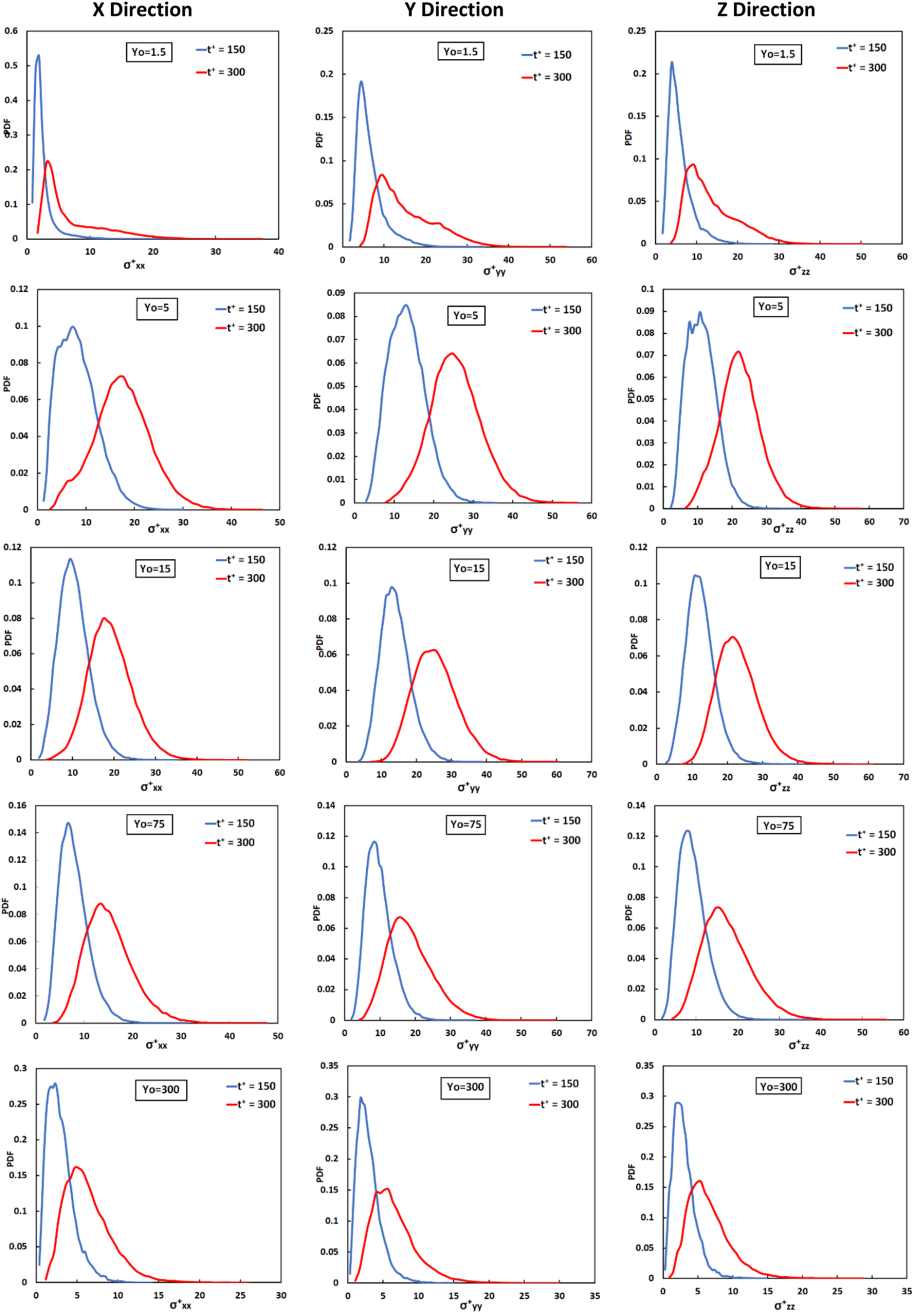
Probability density function of the extensional stress histories in *x*, *y*, *z*, directions for particles released at different positions at t^+^ = 150 and 300 in the Poiseuille flow field.

Figure 8 is a presentation of the PDF of the distribution of the history of the stress on particles at two time instances after being released in the flow field. The PDFs shown are for particles released at Y_0_ = 1.5, 5, 15, 75, 300. It is clear that the integrated stress on particles at t^+^ = 150 shows a narrow distribution, roughly in the range from 0 to 10 for Y_0_ = 1.5 and 300 and from 0 to 25 for Y_0_ = 5, 15, 75. This observation can be interpreted when one considers the Eulerian stress profile depicted in Fig. 5 and the high *Sc* for the vWF particles. For particle release close to the wall of the channel one would expect that a high *Sc* particle would have small diffusion both by molecular means (the standard deviation of the Brownian motion displacement is very low) and by turbulent convection (the turbulent velocity fluctuations that can carry vWF particles away from the wall are very small for y^+^ between 0 and 1.5). At the channel center, as seen in Fig. 5, the average stresses did not vary much between a quarter of the channel height (which is the edge of the log-layer and the beginning of the outer region of the flow, at y^+^ = 150) and the channel center at y^+^ = 300. For release at other locations, in the buffer layer and the viscous sublayer, the vWF particles could diffuse by molecular means and mostly by turbulent convection. In this way they could disperse in the wall-normal direction *y* and would experience a variety of stress values, leading to wider distributions of stress. At later times, as expected, the peaks of the stress history PDFs shifted to the right and the PDFs widened, while the stress distributions tended to be less skewed. As the time of exposure increased, the combined effect of time and stress would accumulate on the particles. As was the case for PC flow discussion for Fig. 4, the width of the distribution is an indication of the significant differences in the stress that particles underwent, and these differences grow as time passed. Note that for any distribution there are particles that experienced a history of low stress (those represented by the tail of the PDF to the left, e.g., the left quartile of the PDF) and others that expected much higher stress history (those represented by the tail of the PDF to the right, e.g., the right quartile of the PDF). In most cases presented in Fig. 8, the right quartile (the top 25% of the particles) see about three times higher stress history than the bottom 25% of the particles.

### Comments on the limitations of the model

Experimental data for the validation of the PDF of the stresses experienced by the vWF surrogate particles in turbulent flow are not readily available. This type of measurement would be non-trivial, especially in a Lagrangian sense (i.e., to mark a particle and measure the stress exerted on it through a turbulent flow in space and time and in three dimensions). Local extensional stresses depend on velocity derivatives that are very noisy to measure (see Eq. 4). One could develop experiments in which to explore the configuration of the vWF after running through velocity fields that look like the ones generated here. This would have led to the validation of the results, if the molecular configuration had been modified on a percentage of the vWF molecules as predicted by our PDF data. Prior experiments, as in^21,79^ could be designed, to control the stress and exposure time to these stresses, but modifications of that apparatus would need to be made to control for elongational stress rather than shear stress (e.g., a parabolic channel flow or a four-roll Taylor mill, instead of Taylor-Couette viscometer)^80^.

The goal of the present study is not to elucidate the molecular mechanism of protein unfolding, and the approach employed in our work cannot offer molecular level information, such as molecular dynamics simulations or coarse-grained simulations can^76,81,82^. In such computations the vWF was modeled taking into account the chain-like structure of the vWF and details of the unfolding of the A1 and A2 domains and the ADAMTS-13 cleavage could be observed, but the simulations were focused on the investigation of one vWF molecule. The approach herein has the advantage of providing the stresses in different flow fields, so that one could potentially use results such as those in refs^76,81^ to determine the critical stress for protein unfolding and to determine what percentage of the proteins in the flow are subject to that stress and for how long. Here, the protein was treated as a particle with a diffusivity that depends on its average radius. Having said this, the trajectories of modified proteins in our model could be calculated by updating the protein diffusivity. If the changes were small (within an order of magnitude), then the results that were obtained (i.e., the probability density functions for stresses, and the history of these stresses along the trajectories of the particles) would be expected to be affected very little. For example, the *Sc* for particles with twice the diameter would not generate significantly different Brownian motion effects, when the *Sc* is already as high as it is here (the Brownian motion is affected by the square root of the *Sc*). Prior work from our laboratory regarding the dispersion of high *Sc* particles in turbulent flow has shown that the behavior of particles with *Sc* > 2400 can be considered as very similar in the statistical sense (e.g., the average velocity of particles released from the same elevation, the values of the Lagrangian autocorrelation coefficient, and the rate of dispersion^49,65^). The trajectories of the particles tracked in the present work are representative of vWF particles only based on the diffusivity of these particles as calculated with the Stokes–Einstein relationship. Solid particles, or other protein molecules of the same diffusivity would follow (according to the modeling assumptions) similar trajectories and would undergo similar levels of stresses in the statistical sense, i.e., the distribution of stresses on any such particles released in these flow fields at the same locations would be subject to the same probability distributions for stresses. Blood cells could be tracked in the same way, but since blood cells are much larger than vWF the equation of motion would need to be modified to account for hydrodynamic drag forces and possibly for added mass terms.

## Conclusions

In this study, direct numerical simulations and Lagrangian scalar tracking methods were applied to compute the extensional stress of vWF surrogate particles in different flow configurations (plane Poiseuille–Couette and plane Poiseuille). The vWF particles were assumed to be spherical, in order to obtain a diffusion coefficient. The changes in the shape of the vWF under stress were not taken into account in the calculation of the diffusivity. The drag forces were assumed to be negligible, while Brownian motion was assumed to be the most dominant contributor to the vWF movement after convection. Following this approach, the three components of the extensional stress were monitored for individual vWF particles over time, enabling calculations of the detailed statistical distributions of the stresses. Such calculations are not trivial for either experimental approaches or computational approaches using turbulence modeling with other low-order numerical methods. On the other hand, these computations are not designed to provide structural information for the vWF or for vWF interactions, such as such as end-to-end-distance of the protein molecule or adherence and adsorption.

The results of the simulation show that the distribution of stress was a function of both time and release position. It was found that both flow conditions lead to a percentage of vWF particles experiencing damaging stresses. The most unpredictable cases were the ones when the vWF was released in the viscous sublayer or in the buffer region of the turbulent flow field. In addition, it is highly probable that particles were exposed to different stresses as they dispersed in the flow field, and these stresses were potentially damaging for a portion of the time they spent in the flow. Injections at the center of the flow field, rather than closer to the wall, would be preferred, as they result in exposure to lower and more predictable stress.

The time history of the stress distribution revealed a similar picture. There are stochastic effects that determine the stress that different particles experience, and the history of stress exposure can vary by multiple times depending on the trajectories of each particle. One would need to develop a power law model for vWF damage, in analogy to power law models for cell trauma^32^, but the exponents of the power law model (Eq. 1) would need to be determined with experimental methods or molecular level computations (e.g., molecular dynamics or coarse-grained techniques). Finally, it is apparent that extensional stresses, which can cause blood clotting or damage to the vWF functionality at levels lower than the shear stresses, should be accounted in predictions of AcWF syndrome, and the whole distribution of stresses should be determined when designing cardiovascular devises. Future research should include the development of stochastic models for estimating the probability of the vWF to be in regions of high or low extensional stresses in turbulent flow fields.

The trajectories of the particles tracked in the present work are representative of vWF particles only based on the diffusivity of these particles as calculated with the Stokes–Einstein relationship. Solid particles, or other protein molecules of the same diffusivity would follow (according to our modeling assumptions) similar trajectories and would undergo similar levels of stresses in the statistical sense, i.e., the distribution of stresses on any such particles released in these flow fields at the same locations would be subject to the same probability distributions for stresses.
